# Supplementary material for: Domestication over Speciation in Allopolyploid Cotton Species: A Stronger Transcriptomic Pull
Source: Genes (Basel). 2023 Jun 20;14(6):1301. doi: 10.3390/genes14061301 (PMC10298526; doi:10.3390/genes14061301)
Supplement: Supplementary file 1 [file genes-14-01301-s001.zip › Supplementary Figure Legends.pdf]

Supplementary Figure S1. PCA of expression data for all samples (A) and for only *G. barbadense* (B). Colors reflect DPA, shapes reflect condition (i.e., wild or domesticated), and AD1/AD2 references to *G. hirsutum* (AD1) and *G. barbadense* (AD2), respectively.

Supplementary Figure S2. UpSet plots comparing DGE overlaps in wild and domesticated accessions between *G. hirsutum* (AD1) and *G. barbadense* (AD2) for 5 DPA (a), 10 DPA (b), 15 DPA (c), and 20 DPA (d).

Supplementary Figure S3. Eigengene expression values for ME4 and ME5 for the *G. hirsutum* (AD1) and *G. barbadense* (AD2) wild (w) and domesticated (d) networks. Colors correspond to DPA, which are ordered from left-to-right as 5 DPA (red), 10 DPA (green), 15 DPA (blue), and 20 DPA (purple).
