# Supplementary figures and images for: Domestication over Speciation in Allopolyploid Cotton Species: A Stronger Transcriptomic Pull
Source: Genes (Basel). 2023 Jun 20;14(6):1301. doi: 10.3390/genes14061301 (PMC10298526; doi:10.3390/genes14061301)

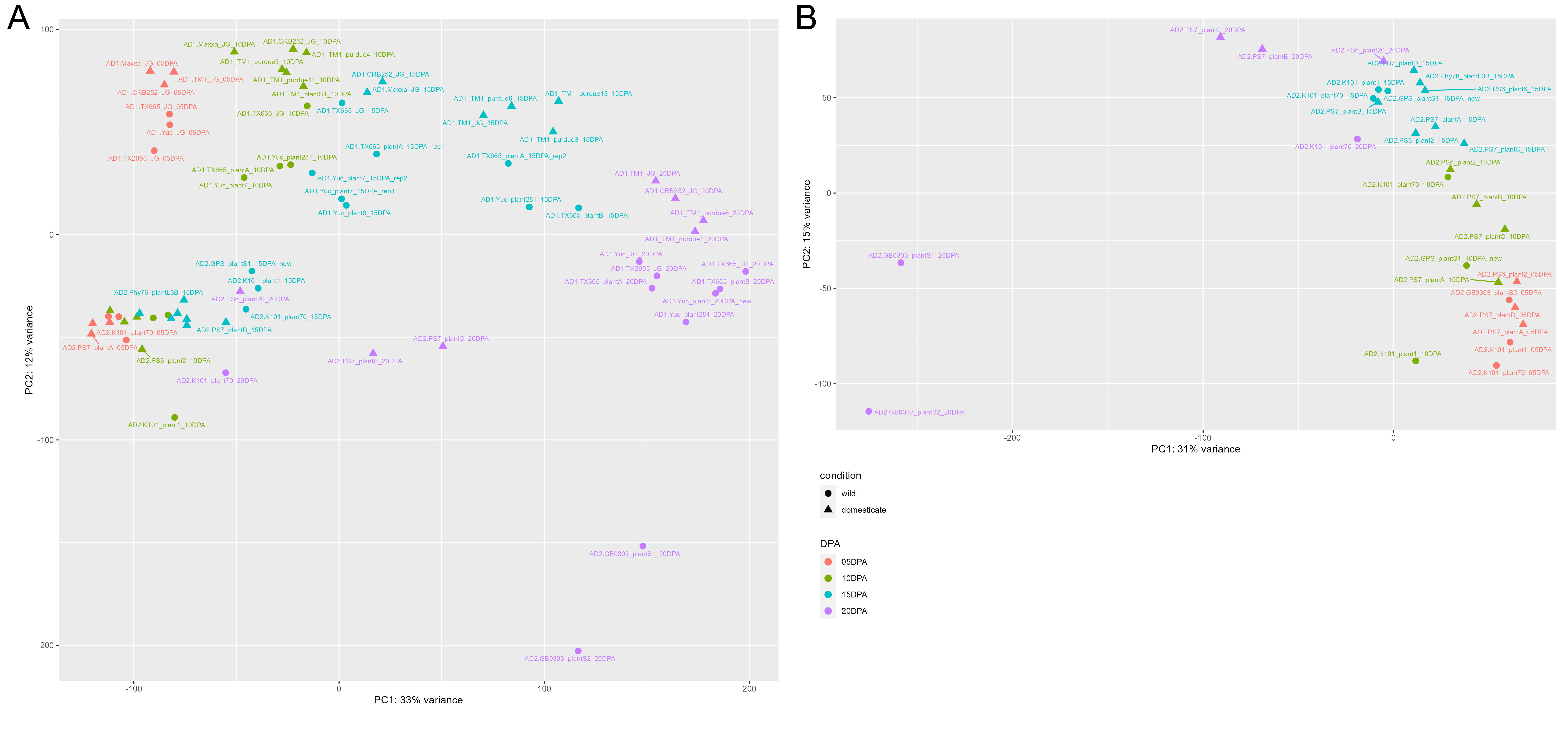

Supplement: Supplementary file 1 [file genes-14-01301-s001.zip › Supplementary Figure S1.jpg]

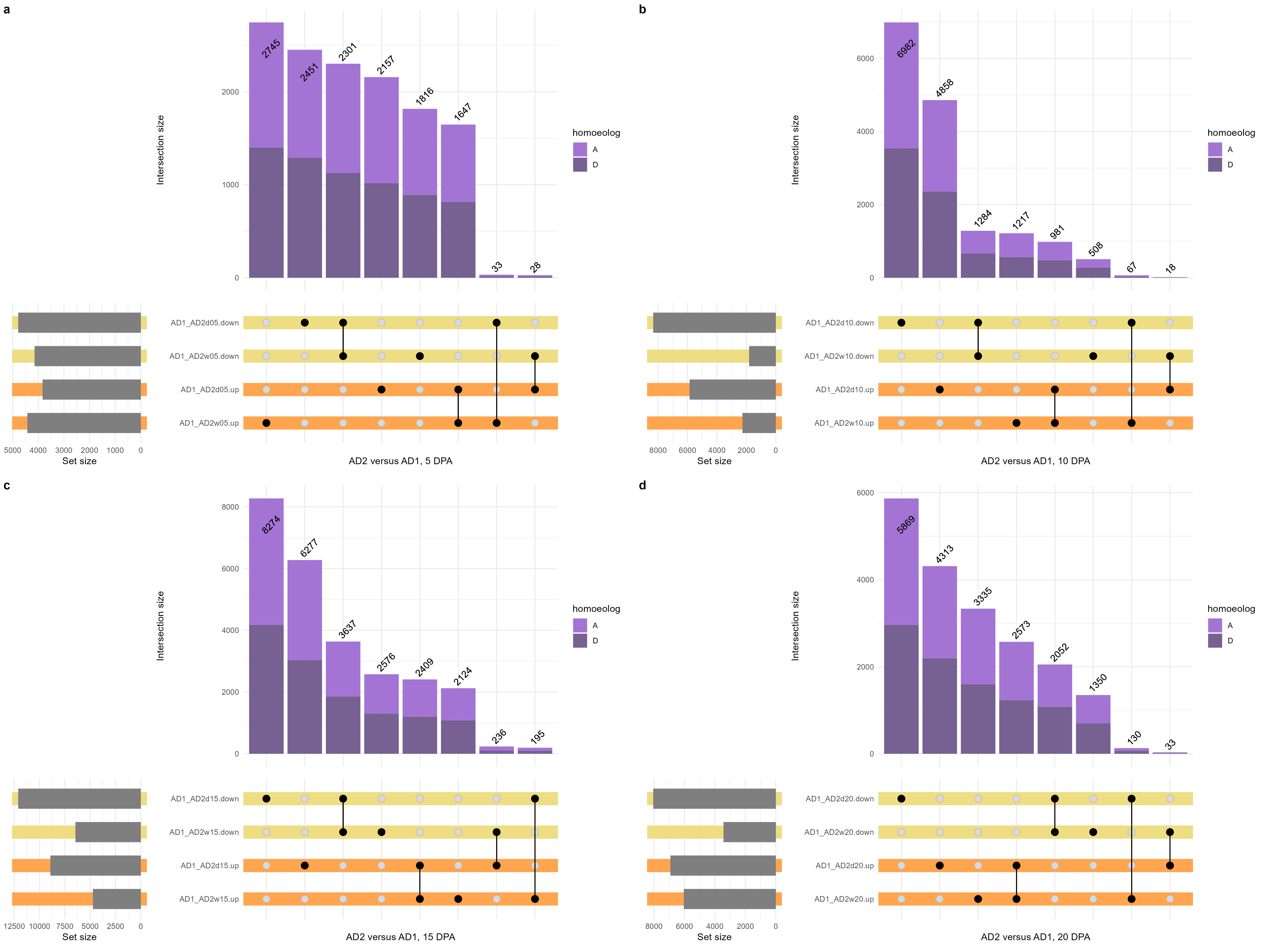

Supplement: Supplementary file 1 [file genes-14-01301-s001.zip › Supplementary Figure S2.jpg]

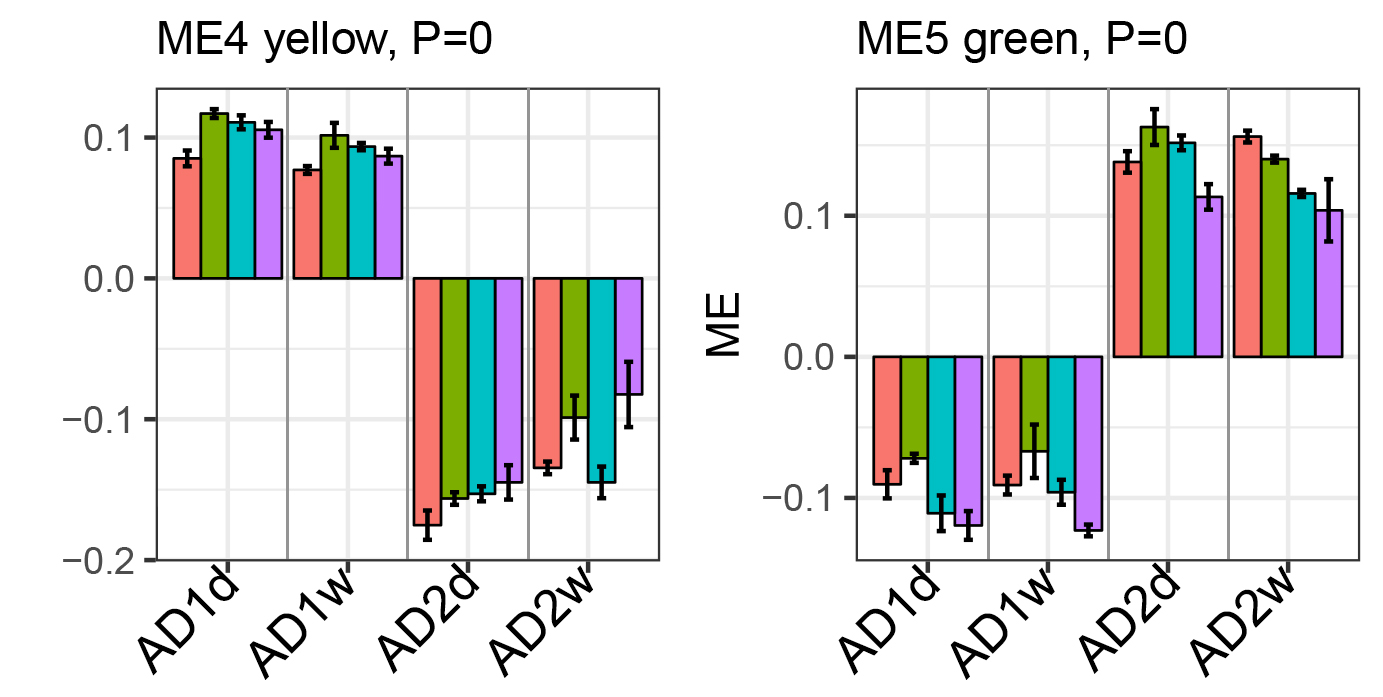

Supplement: Supplementary file 1 [file genes-14-01301-s001.zip › Supplementary Figure S3.jpg]
